# Integrated Analysis of Gene Expression and Tumor Nuclear Image Profiles Associated with Chemotherapy Response in Serous Ovarian Carcinoma

**DOI:** 10.1371/journal.pone.0036383

**Published:** 2012-05-08

**Authors:** Yuexin Liu, Yan Sun, Russell Broaddus, Jinsong Liu, Anil K. Sood, Ilya Shmulevich, Wei Zhang

**Affiliations:** 1 Departments of Pathology, The University of Texas MD Anderson Cancer Center, Houston, Texas, United States of America; 2 Departments of Gynecologic Oncology and Reproductive Medicine and Cancer Biology, The University of Texas MD Anderson Cancer Center, Houston, Texas, United States of America; 3 Department of Pathology, Tianjin Medical University Cancer Institute and Hospital, Tianjin, China; 4 The Institute for Systems Biology, Seattle, Washington, United States of America; Duke-National University of Singapore Graduate Medical School, Singapore

## Abstract

**Background:**

Small sample sizes used in previous studies result in a lack of overlap between the reported gene signatures for prediction of chemotherapy response. Although morphologic features, especially tumor nuclear morphology, are important for cancer grading, little research has been reported on quantitatively correlating cellular morphology with chemotherapy response, especially in a large data set. In this study, we have used a large population of patients to identify molecular and morphologic signatures associated with chemotherapy response in serous ovarian carcinoma.

**Methodology/Principal Findings:**

A gene expression model that predicts response to chemotherapy is developed and validated using a large-scale data set consisting of 493 samples from The Cancer Genome Atlas (TCGA) and 244 samples from an Australian report. An identified 227-gene signature achieves an overall predictive accuracy of greater than 85% with a sensitivity of approximately 95% and specificity of approximately 70%. The gene signature significantly distinguishes between patients with unfavorable versus favorable prognosis, when applied to either an independent data set (*P* = 0.04) or an external validation set (*P*<0.0001). In parallel, we present the production of a tumor nuclear image profile generated from 253 sample slides by characterizing patients with nuclear features (such as size, elongation, and roundness) in incremental bins, and we identify a morphologic signature that demonstrates a strong association with chemotherapy response in serous ovarian carcinoma.

**Conclusions:**

A gene signature discovered on a large data set provides robustness in accurately predicting chemotherapy response in serous ovarian carcinoma. The combination of the molecular and morphologic signatures yields a new understanding of potential mechanisms involved in drug resistance.

## Introduction

Ovarian carcinoma (OvCa) remains a leading cause of mortality from gynecologic cancer, with approximately 21,880 new cases and 13,850 deaths estimated in the United States in 2010 [1], [2]. The standard treatment protocol for advanced-stage epithelial OvCa is cytoreductive surgery followed by platinum-based combination chemotherapy. However, the majority of patients eventually relapse with generally incurable disease, mainly due to the emergence of chemotherapy resistance [3], [4]. Early identification and differentiation of patients who are resistant to chemotherapy could lead to their enrollment in clinical trials with alternative therapeutics and is of utmost importance for improving the outcome of ovarian cancer.

Understanding the molecular mechanisms for chemoresistance has been the subject of intense research. Various genomic methodologies [4]–[12] have been applied to the study of OvCa to identify a gene signature associated with chemotherapy response [6], [13]. However, there is a lack of overlap between the discovered genes in different studies [6], [14], possibly because of limited sample size in most studies.

The Cancer Genome Atlas (TCGA), a project of the National Cancer Institute and the National Human Genome Research Institute, generates a comprehensive catalog of genomic abnormalities with large-scale data sets that include cancers with the highest mortality rates including serous OvCa. In addition, the TCGA effort has led to the accumulation of a large set of tumor images in the repository. It is recognized that cell morphologies are intimately linked to multiple cell functions, such as cell growth, apoptosis, differentiation, and migration [15]–[17]. Switches between different cell functions can be controlled by regulating cell shapes [18], [19]. It is reported that nuclear size is correlated with tumor prognosis in Stage III-IV ovarian cancer [20] and is capable of distinguishing low- from high-grade serous OvCa [21]. However, the molecular mechanism underlying this association remains unknown. The tumor image collections in TCGA provide the opportunity to systematically characterize the morphologic features associated with chemotherapy response and gene activity.

In this study, we leverage the full scope of the TCGA database with a large population of patients, including gene expression and tumor images, to identify the molecular and morphologic signatures associated with chemotherapeutic response in OvCa. Integration of the genomic and morphologic dimensions of OvCa will yield potential insights into mechanism of drug resistance and facilitate identification of novel system-level events for alternate therapeutic interventions.

## Results

### Gene Signature Associated with Chemotherapy Response in Serous OvCa

**Figure 1:**
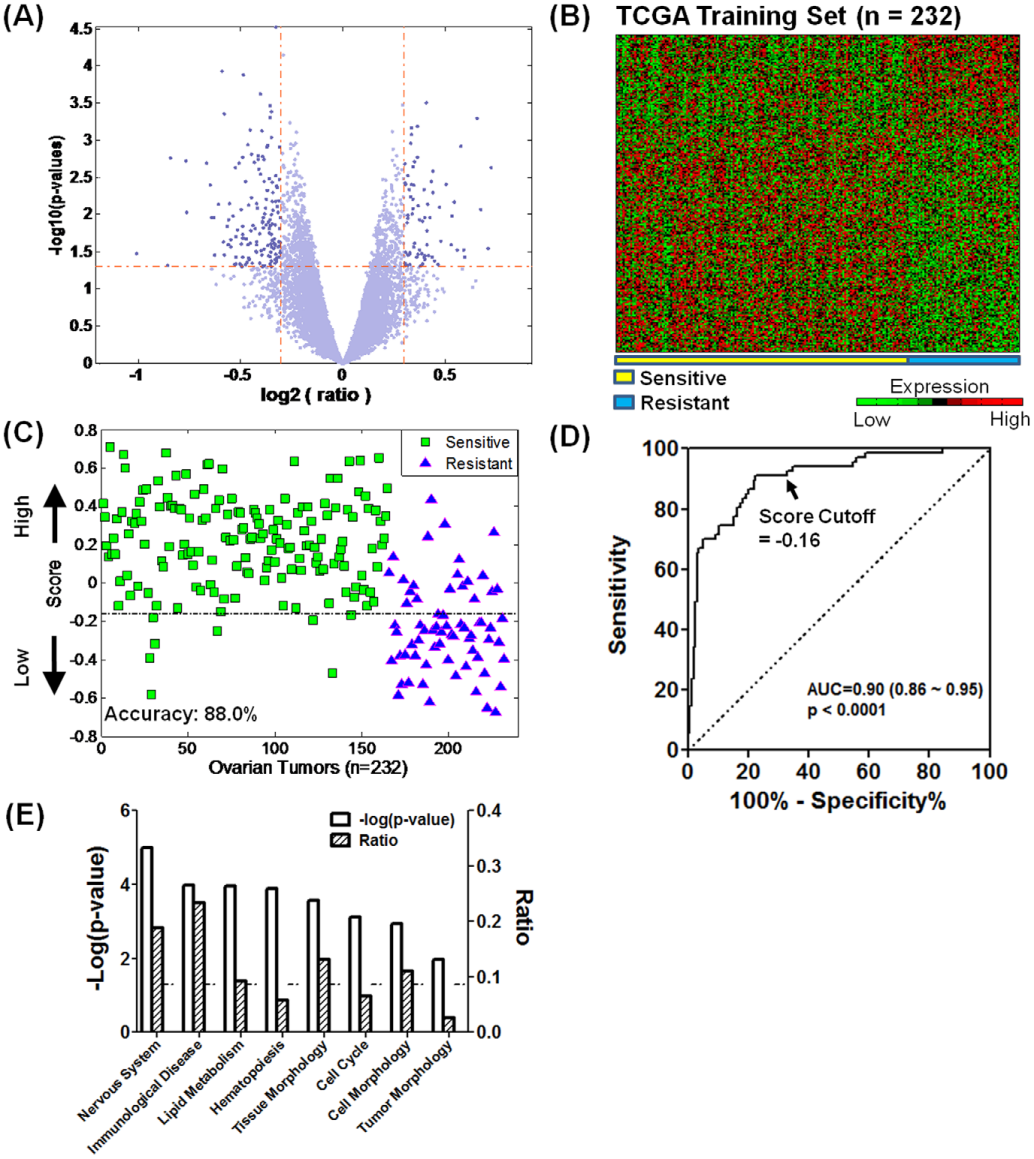
Gene signature associated with chemotherapy response in serous OvCa. (A) Identification of gene signature differentially expressed in chemoresistant and chemosensitive patients. (B) The selective genes (n = 227) distinguish the chemoresistant patients from the chemosensitive patients. (C) A predictive model on the basis of the gene signature reveals an accuracy of approximately 87.9% in correctly classifying chemoresistant and chemosensitive tumors (n = 232; green square = chemosensitive, blue triangle = chemoresistant). (D) An receiver operating characteristic (ROC) curve illustrates the predictive performance, with a sensitivity of approximately 95.2% and specificity of approximately 70% at the predictive score cutoff of approximately −0.16 that serves as a threshold for patient stratification in the TCGA data set. AUC: area under curve. (E) Pathway analysis shows that the gene signature is enriched in the morphologic function at cellular, tissue, and tumor levels. The dotted line denotes the cutoff for significance (*P* = 0.05). The shaded bars show the ratio of genes enriched in each function to the 227 genes.

To gain insight into the potential mechanisms underlying the differential response of OvCa to chemotherapy, we perform an integrated analysis of gene expression and tumor nuclear image profiles. The 232-sample set that has both gene expression and image data is used to identify a gene expression pattern that could predict clinical outcome. The 227 genes most weighted in achieving the prediction are identified, of which 154 (67.8%) were downregulated and 73 (32.2%) are upregulated in the chemoresistant group (Figure 1A; Table S1). The gene expression fold change cutoff between these two groups is determined on the basis of the overall predictive accuracy of the patients in this training set (Figure S1); this cutoff is similar to the one used in the previous study [9]. The gene expression profiling well separates the chemoresistant patients from the chemosensitive patients (Figure 1B) and achieves an overall predictive accuracy of approximately 87.9% (Figure 1D), with a sensitivity of approximately 95.2% and specificity of approximately 70% (Figure 1C). Pathway analysis of the discovered genes reveals an enrichment of several groups of genes that regulate morphologic changes at the cellular (approximately 11%), tissue (approximately 13%), and tumor (approximately 3%) levels (Figure 1E).

### Validation of Gene Signature

**Figure 2:**
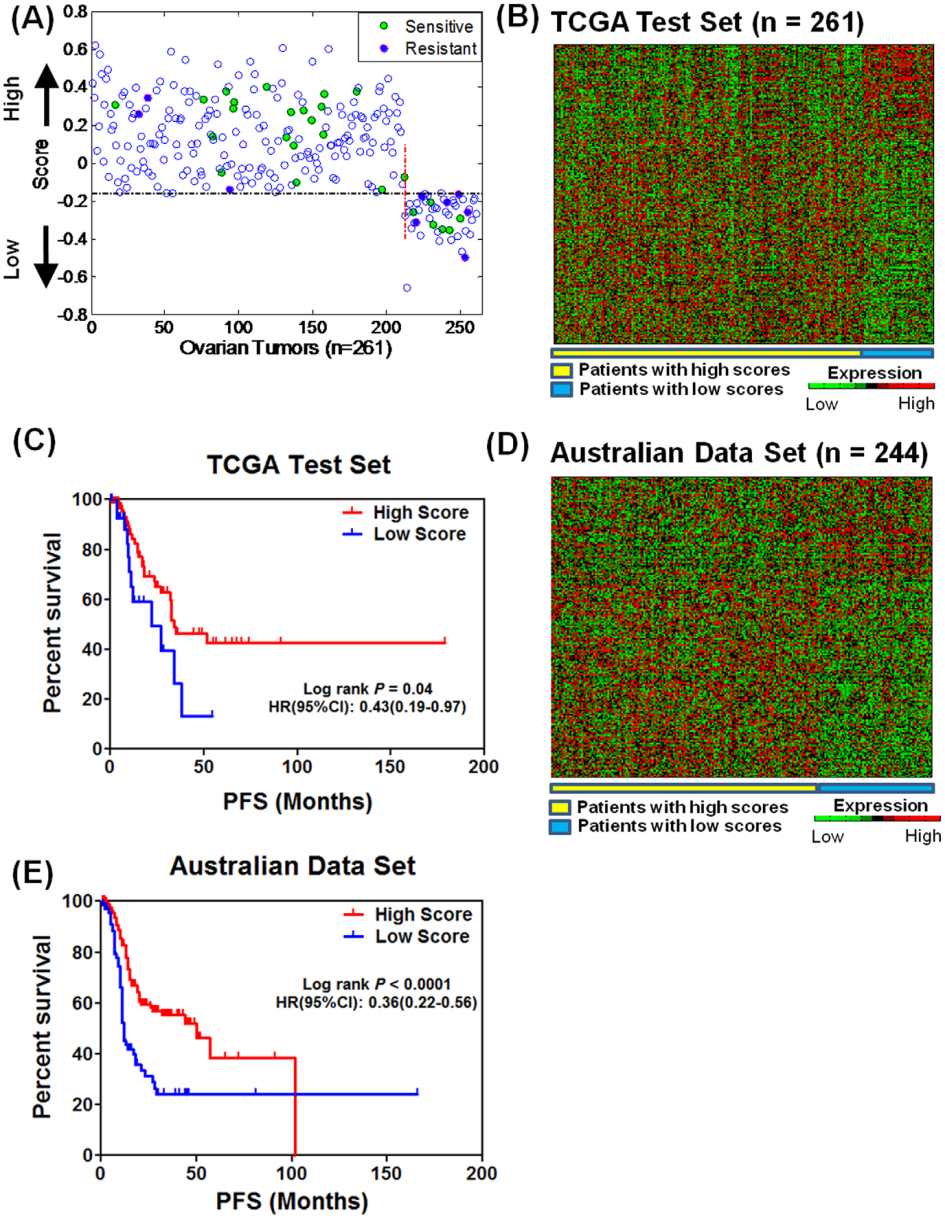
Validation of gene signature. (A) The predictive model constructed from the TCGA training set was applied to an independent TCGA validation set (n = 261) and split the patients into two groups based on the score cutoff of −0.16 as determined by the ROC curve. Thirty five patients are identified to have an explicit response to chemotherapy [22]. (B) The two groups are well separated, with 212 patients in the low-scoring group and 49 in the high-scoring group. (C) Exclusion of patients with no survival data resulted in 109 patients in the low-scoring group and 29 in the high-scoring group. Kaplan-Meier analysis shows patients in the high-scoring group had poorer progression-free survival (*P* = 0.04). (D) The predictive model as applied to the external data set distinguishes the patients in the low-scoring group from in the high-scoring group; where the low-scoring group consists of the 70.1% patients (171 out of 244) with the highest predictive scores, and the high-scoring group consists of the 29.9% patients (73 out of 244) with the lowest predictive scores (see text for details). (E) Kaplan-Meier analysis shows patients in the high-scoring group had poorer progression-free survival than those in the low-scoring group (*P*<0.0001).

A validation of the gene signature is performed on an independent set of 261 samples from TCGA. Based on the score cutoff from Figure 1D, the predictive model splits the patients into two groups (Figure 2A) that are well separated from each other (Figure 2B). 35 patients are identified to have an explicit response to chemotherapy [22] and 26 of them are correctly predicted (Figure 2A). Kaplan-Meier analysis of the remaining samples after removing the patients without survival data shows that the patients in the low-scoring group exhibited poorer progression-free survival (PFS) (Figure 2C; median: 22.3 vs 34.2 months; log-rank *P* = 0.04, HR [95%CI] = 0.43 [0.19–0.97]). The clinicopathologic characteristics of patients in these two groups are summarized in Table S2.

Robustness and scalability of the gene signature are next evaluated by using the Australian data set which is based on a different microarray platform. We use it to validate whether the discovered genes are associated with patient outcome. Overlap analysis reveals 198 among the 227 genes in this data set (Table S1). Using the threshold from the reported chemosensitive rates in ovarian cancer patients (approximately 70% [6]), we group the ∼70.0% patients (171 out of 244) with the highest scores into the high-scoring group and the remaining 30% patients (73 out of 244) into the low-scoring group (Figure 2D); consistently patients in the low-scoring group have poorer prognosis (Figure 2E; *P*<0.0001, HR [95%CI] = 0.36 [0.22–0.56]) where the median PFS of Group 2 (12.0 months) is almost 4 times shorter than that of Group 1 (50.0 months). The clinicopathologic characteristics of the patients in these two groups either with high scores or with low scores in both validation sets are detailed in Table S2, which shows the similar age, tumor stage, and tumor grade distributions as the TCGA training set (Table 1). Cox proportional hazard analysis demonstrates that the two groups have significantly different progression-free survival patterns, independent of age, grade, and stage (Table S3).

**Table 1:** Clinicopathologic characteristics of TCGA patients with serous OvCa that are used for tumor nuclear image profile and gene expression profile analyses.

	TCGA Cohort		
	Clinical Chemosensitive	Clinical Chemoresistant	Totals (All)
**No. of patients**	172	81	253
**Age**			
**Mean, yrs [SD]**	59.1 [11.4]	61.7 [11.0]	59.9 [11.4]
**Range**	30.5–87.5	38–84.7	30.5–87.5
**FIGO Stage** ¶			
**II**	13	0	13
**III**	134	69	203
**IV**	25	12	37
**WHO Grade**			
**2**	29	8	37
**3**	139	72	211
**Unknown**	4	1	5
**Surgical outcome** ξ			
**Optimal (≤1 cm)**	70	48	118
**Suboptimal (>1 cm)**	43	19	62
**No macroscopic disease**	40	9	49
**Unknown**	19	5	24
**Vital status**			
**Alive**	80	14	94
**Dead**	91	67	158
**Unknown**	1	0	1
**Recurrent disease** ζ			
**Yes**	144	81	225
**No**	28	0	28

Abbreviations: FIGO, International Federation of Gynecology and Obstetrics; TCGA, The Cancer Genome Atlas; SD, standard deviation; WHO, World Health Organization.

¶ : Cases were staged according to the 1988 FIGO staging system.

ξ : Surgical outcome was defined as the size of residual disease at the conclusion of the primary surgical procedure. This field was used to define surgical cytoreduction as optimal or suboptimal. Optimal was defined as no residual disease greater than 1 cm and included the variable categories of no macroscopic disease (*i.e.* microscopic residual disease) and 1 to 10 mm. Suboptimal was defined as residual disease greater than 1 cm and included the variable categories of 11 to 20 mm and greater than 20 mm.

ζ : Local recurrence after the date of initial surgical resection.

These results not only validate the predictive performance of the gene signature but also suggest its strong association with tumor prognosis, which is most likely contributable from chemotherapy response.

### Tumor Nuclear Image Profile Associated with Chemotherapy Response in Serous OvCa

**Figure 3:**
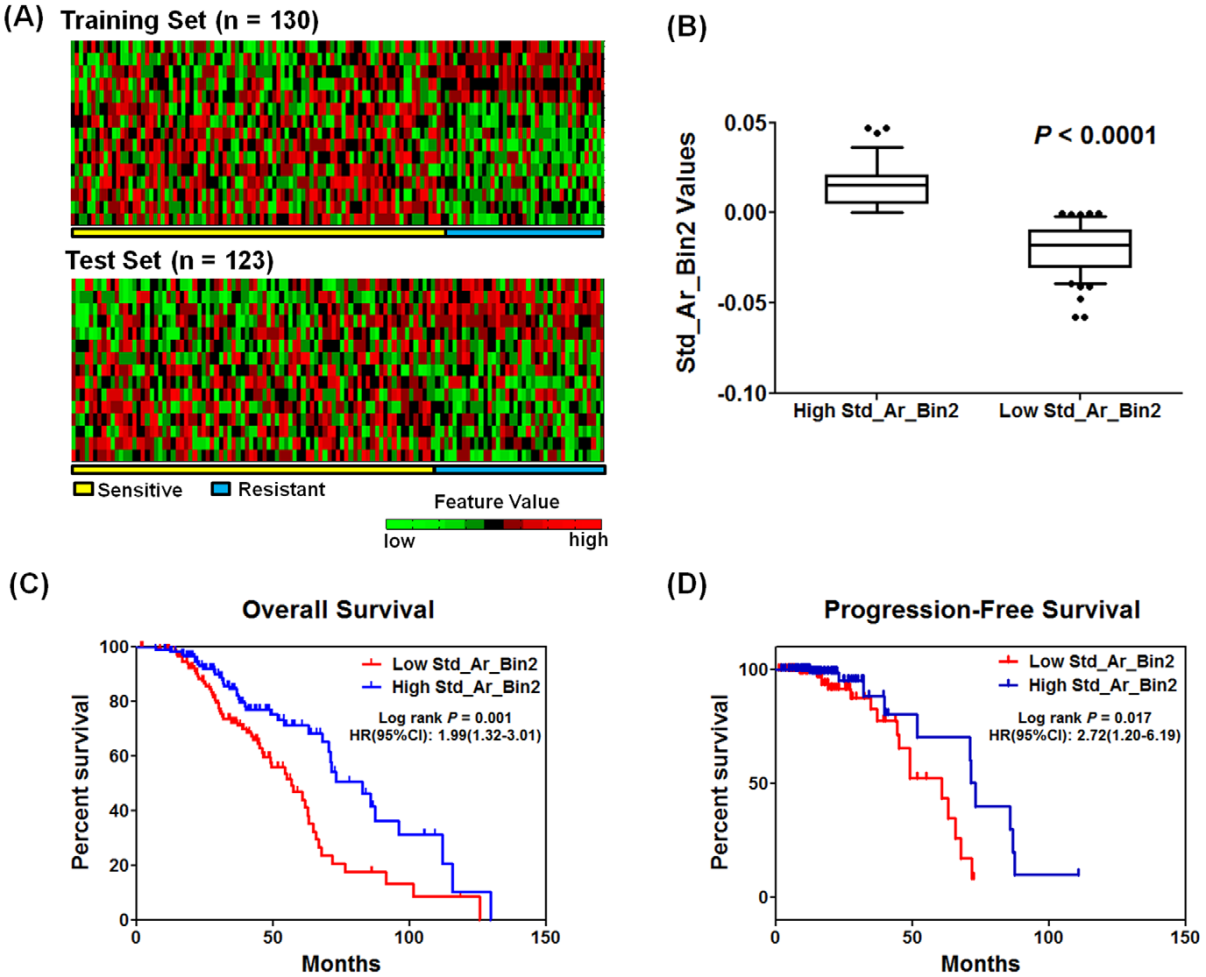
Tumor nuclear image profile associated with chemotherapy response. (A) The tumor nuclear image profile demonstrates a strong association with chemotherapy response in both the training set (top) and the validation set (bottom). Each row corresponds to a morphologic feature, with the columns corresponding to data in different samples. Feature values were median centered across the tumor set and then log transformed. A detailed version of this panel with morphological feature names is provided in the Figure S5. (B) Feature (Std_Ar_Bin2) distribution across the entire image sample set (n = 253) where patients with values greater than or equal to the feature median are categorized into a group (i.e., High Std_Ar_Bin2, n = 129), and patients with values less than the feature median are categorized a different group (i.e., Low Std_Ar_Bin2, n = 124). Smaller values of Std_Ar_Bin2 feature are significantly associated with poorer OS (C) and poorer PFS (D).

The results from pathway analysis (Figure 1E) suggests that the morphologic characteristics may play a key role in determining chemotherapy response. The tumor nuclear image profile in the 130-sample training set is used to identify a morphologic signature associated with chemotherapy response; this is then validated using the 123-sample validation set. The 15 significant features (*FDR*≤2%, Table S4) consisting of 5 with the highest signal-to-noise ratios (SNRs) and 10 with the smallest SNRs clearly uncovers binary patterns in both the training and validation sets, as illustrated in a similar fashion commonly used in gene expression profiles (Figure 3A). A detailed version of this panel with morphological feature names is provided in the Figure S5. More prominently, we find that the Std_Ar_Bin2 feature (see Methods and Table S5) values are strongly associated with tumor prognosis. Based on the Std_Ar_Bin2 feature values, we split the 253 patients into two groups, where patients with values greater than or equal to the median are categorized into a group (ie, High Std_Ar_Bin2, n = 129), and patients with values less than the feature median are categorized a different group (ie, Low Std_Ar_Bin2, n = 124) (Figure 3B). Kaplan-Meier survival analysis demonstrates that tumors with smaller values of Std_Ar_Bin2 feature have significantly poorer overall survival (OS) (Figure 3C, log-rank *P* = 0.001, HR [95%CI] = 1.99 [1.32–3.01]) and poorer PFS (Figure 3D, log-rank *P* = 0.017, HR [95%CI] = 2.72 [1.20–6.19]). Cox proportional hazard analysis demonstrates that the two subgroups, split on the basis of Std_Ar_Bin2 feature values, have significantly different OS and PFS patterns, after controlling for age, stage, and grade (Table S6). These results suggest that morphologic features are significantly related to patient survival and could serve as valuable prognostic markers [23].

### Integrated Analysis of Morphologic Features and Gene Signature

**Figure 4:**
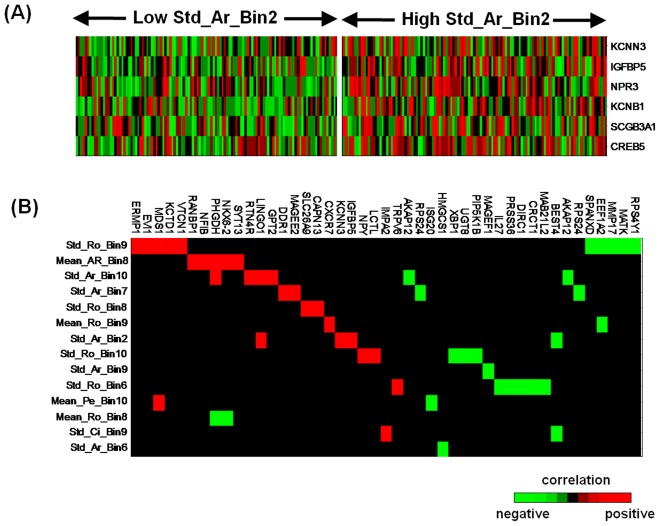
Integrated analysis of morphologic features and gene signature. (A) Supervised analysis of gene expression data on the patients split by the Std_Ar_Bin2 feature values as described by Figure 3B. (B) Correlation of the highly correlated feature-gene pairs (*P*<0.005), with negative correlations in green and positive correlations in red.

Both genomic and morphologic features are associated with chemotherapy, suggesting that these two types of signatures are strongly associated with each other. With the patients split into the two groups based on the Std_Ar_Bin2 feature values, as described above, we carry out a supervised analysis of the gene expression data and find that five of the signature genes are significantly lower (*P*<0.01) in the Low Std_Ar_Bin2 group (Figure 4A). Similar analysis is performed on the other morphologic features, and the corresponding differentially expressed genes are summarized in Table S7. Next we perform correlation analysis of morphologic feature data and gene expression data, and the highly correlated (either positively or negatively) feature-gene pairs (*P*<0.005) are depicted in Figure 4B, in which we can see that the morphologic features are strongly related to the gene signature.

## Discussion

Several studies have described chemotherapy response in ovarian cancer using gene expression profiles, as summarized by Helleman *et al* [14]. However, the number of ovarian cancer specimens used for the gene selection in those studies was relatively small, ranging from 6 to 119, and the corresponding gene sets discovered to be associated with platinum-based chemotherapy resistance exhibited a wide range of 14 to 1,727 genes where only seven genes were observed as an overlap and each between only two gene sets [14]. Lack of overlap between the discovered gene sets is likely due to the limited sample size in most studies. However, ours is the first study performed on such a large scale, two genes in the 227-gene set, EPH receptor B3 (EPHB3) and nuclear factor I/B (NFIB), had been identified in one of the previous studies [12], and one gene, RNA binding protein 1 (RNABP1), had been identified in a different study [24]. More prominently, a gene set discovered on a large data set undoubtedly has high statistical power and robustness in accurately predicting chemotherapy response. Recently, the TCGA research network identified 193 prognostic gene signatures predictive of OS, but the gene association with chemotherapy response remains unexplored [22]. Here we used a large sample set (493 samples from TCGA and 244 samples from an external source) for identification of molecular and morphologic signatures that are associated with chemotherapy response. The predictive model on the basis of gene signature revealed an accuracy of 87.9% in correctly classifying refractory from responsive tumors in the TCGA training set and stratified patients in both the TCGA validation set and the Australian data set into groups that demonstrated significant discrepancy in tumor progression, suggesting the capacity of the gene signature to serve as a mechanism to stratify patients with respect to treatment.

The imaging approach stratifies the cells into 10 bins based on nuclear size and accounts for the heterogeneity of cells in a tumor population. Our stratification revealed that most significant morphologic features differed between the chemosensitive and chemoresistant groups in the larger nuclei (range, 300 to 500 pixel^2^; Table S2). However, nuclei within this size range account for a very small percentage (approximately 2.0%), and the majority of the nuclei (approximately 98.0%) do not show a significant difference in chemotherapy response. This observation not only is consistent with the Goldie-Coldman hypothesis [25] that only a small cell population may contribute to differential response to chemotherapy, but also suggests the difficulty of a conventional approach of simply correlating the overall morphologic differences with chemotherapy response, owing to the “dilution” effect [26]. Therefore, our imaging approach allows us to interrogate different cell populations separated on the basis of nuclear size in a high throughput and automated fashion.

The 15 morphologic features (Table S4) most weighted in achieving the patient separation are highly instructive. The same nuclear parameter might exhibit different or even opposite patterns. The average roundness of nucleus in Bin 8 (Mean_Ro_Bin8) is significantly higher in the chemoresistant group (*P* = 1.5×10^−4^, Figure S2A), on the contrary, the same nuclear parameter in Bin 9 (Mean_Ro_Bin9) shows significant decrease in the chemoresistant patients (*P* = 0.0015, Figure S2B). The average roundness of the entire nucleus per sample (Mean_Ro_Total) shows no significant difference (*P* = 0.56, Figure S2C). In addition, none of the image features calculated from the entire nucleus per sample, the way similar to those used in other studies [20], [21], [26], show significant difference between the chemoresistant and chemosensitive patients. This discrepancy from the previous studies [20] likely results from the number of nuclei used in the feature calculation. We used approximately 4000 nuclei per sample for feature value calculation, almost 80 times more than the amount used in the other studies [20], [21], [26]. Taken together, our approach of binning the nucleus size and then assessing the image feature in each individual bin improves the image feature resolution and enhances the discriminating power. Furthermore, our approach of calculating the morphologic features in separate bins (with smaller size variations) is capable of alleviating the size dependence of some of the features, such as circularity and roundness [27].

Aside from the potentially practical value, the morphologic features also provide insights into cancer morphogenesis. The chemosensitive patients exhibit a smaller value of nuclear roundness in Bin 8 (Mean_Ro_Bin8), but with a larger variability (Std_Ro_Bin8) and a larger aspect ratio (Mean_AR_Bin8). Such morphologic differences likely result from the active response of the cells to their environment and heightened cellular metabolism, that is contributable from different molecular regulations (Figure 4B, Table S7). This is further corroborated by pathway analysis, which revealed the gene enrichment in the morphologic function at cellular, tissue, and tumor levels (Table 2). The gene content of this table offers potential insight into the structural and molecular mechanisms of the chemotherapy response. The importance of A2M gene expression is of particular interest, in view of past work suggesting a correlation between decreased A2M levels with sensitivity to drugs [28]. A2M is an inhibitor of matrix metalloproteinase activity, which is reported to contribute to tissue remodeling and morphogenesis [29], [30]. PAX6, which is associated with drug response, is strongly activated by cotylenin A in retinoblastoma cell lines [31]. Decreased expression of EPHB3 in the chemoresistant group may have promoted chemoresistance by impairing the apoptotic response to cell damage [12].

**Table 2:** Morphologically related genes at cellular, tissue, and tumor levels.

Gene	Entrez Gene Name	Fold difference*	p-value	Location
A2M	alpha-2-macroglobulin	0.81	2.6E-02	Extracellular Space
AQP5	aquaporin 5	0.81	1.1E-02	Plasma Membrane
AREG	amphiregulin	1.51	3.7E-02	Extracellular Space
AVIL	advillin	0.77	6.2E-03	Cytoplasm
CALML3	calmodulin-like 3	1.36	5.0E-03	Cytoplasm
CD38	CD38 molecule	0.71	2.0E-02	Plasma Membrane
CNN2	calponin 2	1.24	1.0E-02	Cytoplasm
CXCR4	chemokine (C-X-C motif) receptor 4	0.80	2.6E-02	Plasma Membrane
DDR1	discoidin domain receptor tyrosine kinase 1	0.81	1.2E-03	Plasma Membrane
DKK1	dickkopf homolog 1 (Xenopus laevis)	1.47	2.9E-02	Extracellular Space
EFNB2	ephrin-B2	0.80	3.8E-02	Plasma Membrane
EPHB3	EPH receptor B3	0.76	1.2E-02	Plasma Membrane
FOXA2	forkhead box A2	0.64	4.0E-03	Nucleus
GAP43	growth associated protein 43	1.33	1.6E-02	Plasma Membrane
GDF6	growth differentiation factor 6	1.32	4.0E-02	Extracellular Space
GFRA1	GDNF family receptor alpha 1	1.31	1.2E-02	Plasma Membrane
HES1	hairy and enhancer of split 1, (Drosophila)	0.80	1.4E-02	Nucleus
SD11B2	hydroxysteroid (11-beta) dehydrogenase 2	0.77	1.8E-03	Cytoplasm
ICAM5	intercellular adhesion molecule 5, telencephalin	1.27	2.4E-02	Plasma Membrane
IGFBP5	insulin-like growth factor binding protein 5	0.75	7.0E-03	Extracellular Space
IGHM	immunoglobulin heavy constant mu	0.66	7.2E-03	Plasma Membrane
IGKC	immunoglobulin kappa constant	0.56	1.7E-03	Extracellular Space
IL15	interleukin 15	1.31	4.6E-02	Extracellular Space
KCNH2	potassium voltage-gated channel, subfamily H (eag-related), member 2	0.78	1.2E-03	Plasma Membrane
LIPG	lipase, endothelial	0.73	7.4E-03	Extracellular Space
MATK	megakaryocyte-associated tyrosine kinase	1.27	2.2E-03	Cytoplasm
MDK	midkine (neurite growth-promoting factor 2)	0.75	2.4E-03	Extracellular Space
EVI1	MDS1 and EVI1 complex locus	0.77	4.6E-03	Nucleus
MMP1	matrix metallopeptidase 1 (interstitial collagenase)	0.69	4.7E-02	Extracellular Space
NPAS3	neuronal PAS domain protein 3	0.70	1.6E-02	Nucleus
NPY	neuropeptide Y	1.64	2.8E-02	Extracellular Space
NRG4	neuregulin 4	0.71	8.5E-03	Extracellular Space
NTF5	neurotrophin 4	1.35	3.7E-02	Extracellular Space
PAX6	paired box 6	0.75	1.8E-02	Nucleus
PCSK6	proprotein convertase subtilisin/kexin type 6	0.81	2.4E-02	Extracellular Space
POU2AF1	POU class 2 associating factor 1	0.63	2.0E-03	Nucleus
POU5F1	POU class 5 homeobox 1	1.24	1.1E-02	Nucleus
RTN4R	reticulon 4 receptor	0.78	3.4E-04	Plasma Membrane
S100A4	S100 calcium binding protein A4	0.78	4.6E-02	Cytoplasm
SLC1A3	solute carrier family 1 (glial high affinity glutamate transporter), member 3	0.81	1.5E-02	Plasma Membrane
SPOCK2	sparc/osteonectin, cwcv and kazal-like domains proteoglycan (testican) 2	1.29	3.4E-02	Extracellular Space
TRPV6	transient receptor potential cation channel, subfamily V, member 6	0.80	6.4E-03	Plasma Membrane
TSPAN7	tetraspanin 7	0.69	1.0E-02	Plasma Membrane
XBP1	X-box binding protein 1	0.80	9.2E-03	Nucleus

* Fold difference in geometric means of chemoresistant tumors (numerator) compared with chemosensitive tumors (denominator).

In conclusion, a gene signature discovered on a large data set provides robustness in accurately predicting chemotherapy response in serous OvCa. Meanwhile, we propose a novel approach for tumor nuclear image profile generation by characterizing patients with nuclear features (such as size, aspect ratio, and roundness etc) in incremental bins, and we demonstrate that the tumor nuclear image profile exhibits a strong association with chemotherapy response. This imaging approach is capable of accounting for cell heterogeneity and improving the discriminating power. The integrated approach herein, using gene expression profile that predicts chemotherapy response coupled with the morphologic features to stratify patients to the most appropriate treatment regimen, represents an important step toward the goal of personalized cancer treatment by identifying the area where novel drugs can be developed. Although our observations suggest that the tumor image profile is capable of defining prognosis and yielding mechanistic insights into the process of chemoresistance, one limitation of this study is the lack of validation of the image analysis due to unavailability of the independent image sets especially in a large population. This issue should be addressed in the future in order to determine the ultimate value of this technique in clinical practice. Besides, the resolution dependence of the morphologic features in separate bins has not been systematically investigated yet in this study and deserves attention in the follow-up studies. Future work also consists of inclusion of more possible morphologic features and verification of the gene-feature relation identified in this study.

## Materials and Methods

### Patients and Tissue Samples

Two hundred fifty three OvCa patients in the TCGA database with explicit platinum status [22] are obtained for nuclear image profile generation, among which 172 patients are sensitive to chemotherapy, and 81 are chemoresistant. Platinum status is defined as resistant if the patient recurred within six months. Platinum status is defined as sensitive if the platinum free interval is six months or greater, there is no evidence of progression or recurrence, and the follow-up interval is at least six months from the date of last primary platinum treatment [22]. Compared with patients who are chemosensitive, the chemoresistant patients exhibit relatively poorer overall survival (OS; median, 53.9 vs. 33.8 months; p<0.0001) and progression-free survival (PFS; median, 25.8 vs. 9.3 months; p<0.0001; Figure S3). Other characteristics of these 253 patients are listed in Table 1. The average age at diagnosis is 61.7 years (range, 38.0 to 84.7 years) for the chemoresistant group and 59.1 years (range, 30.5 to 87.5 years) for the chemosensitive group. Up to 84% of the chemosensitive patients show the symptom of recurrent diseases in contrast to 100% of relapse for the chemoresistant patients. 232 among the 253 samples with expression data serve as the TCGA training set to identify the gene signature, of which 165 are chemosensitive and 67 are chemoresistant. An independent data set from TCGA (n = 261) and an external data set from an Australian study [32] are applied for validation of the gene signature. The gene expression profile in TCGA dataset was performed on three different platforms (Affymetrix Exon 1.0, Agilent 244 K Whole Genome Expression Array and Affymetrix HT-HG-U133A) and a unified expression data set was created by the TCGA research working group and is available in the TCGA data portal. The external expression data was performed on the Affymetrix HG-U133 plus 2 platform and was downloaded from the Gene Expression Omnibus (accession GSE 9899 [32]). The training set is used to discover the gene signature and then to create the predictive model. To be consistent with patient characteristics in these two data sets, we exclude patients from the Australian data set who have either non-serous OvCa or grade 2 disease, resulting in 244 patients in this validation set. The clinicopathologic characteristics of patients in these two validation data sets are summarized in Table S2.

### Genomic Data Analysis

Expression data are prescreened to remove genes with trivial variation across the samples and low median expression levels, resulting in 14,084 genes in the analysis. The gene signature identified through a supervised method [33] is used for constructing a predictive model using the weighted voting algorithm [34], [35]. A predictive score is assigned to each sample and is calculated as
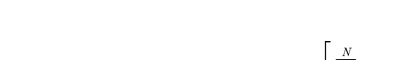
where 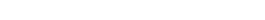, N is the number of discovered genes, w_f_ is the weighting factor, x_f_ is the expression value and μ represents the expression mean for each class. A sample with a score greater than a cutoff is assigned to the chemosensitive group, and a sample with a score less than or equal to the cutoff is assigned to the chemoresistant group. The predictive accuracy, based on a cutoff score determined by receiver operating characteristic (ROC) curve, is assessed. The gene signature is validated on an independent sample set from TCGA and an external data set [32]. Pathway and network analysis is performed using Ingenuity Pathway Analysis (IPA, version 8.6-3003; Ingenuity Systems, Inc.).

### Tumor Nuclear Image Profile Generation and Analysis

#### Nucleus parametric profile generation

An average of 10 high-resolution tumor images (20 X magnification, 1072×648 pixels) per sample at the different views of the tissue blocks are first selected by a pathologist from hematoxylin- and eosin-stained ScanScope virtual slides, to account for the spatial heterogeneity of the tumor tissues. Next, we automatically identify and measure the nuclei in each image by using a cell-image analysis software (ImageJ, version 1.42, NIH) [36], producing a parametric profile for each nucleus. In brief, the first slice of the tumor image is processed using a Fast-Fourier-transform (FFT) band pass filter with the default setting before it is converted to a black-white image (features of interest such as nuclei are displayed as black and the background as white) by using a threshold value verified by overlaying the segmented nuclei with the original RGB image. Nuclei with a size range of 50 to 500 pixel^2^ and a circularity of greater than 0.3 are selected for further analysis. The nucleus profile consists of a set of numbers that describe the nucleus’s characteristics, including size, location, and shapes that are automatically measured using the ImageJ Plugins, which is widely used in research [37], [38]. Definition of these nuclear parameters is described in details in the ImageJ user guide [39]. Typically, approximately 4000 nuclei within the size range from 50 to 500 pixel^2^ per sample are produced which is almost 80 times more than the amount used in other studies [20], [21]. An example detailing this procedure is shown in Figure S4.

#### Tumor image profile generation

To generate the tumor image profile, we first split the nuclei into 10 evenly spaced bins based on the nuclear size, and then calculate the average value and standard deviation (SD) of these parameters (e.g, area, perimeter, circularity, aspect ratio, solidity, and roundness) for each nucleus in each bin as well as for all the nuclei in an image. In addition, the number of nuclei in each bin (feature name, count) and its percentage (feature name, percentage) are defined as image features and calculated accordingly. The compactness is defined as an image feature to qualify the spatial distribution of nuclei within the tumor tissue. As a result, the tumor image profile consists of 153 morphologic features including 66 means, 66 SDs, 10 percentages, 10 counts, and 1 compactness (Table S5). Definition of these nuclear parameters is described in details in the ImageJ user guide [39].

#### Tumor image profile analysis

The 253 TCGA samples with the calculated image profile (described above) are randomly divided into a 130-sample training set and a 123-sample validation set in an approximate 1∶1 ratio. The training set consists of 90 chemosensitive patients (69.2%) and 40 chemoresistant patients (30.8%), while the validation set contains 82 chemosensitive patients (66.7%) and 41 chemoresistant patients (33.3%). The distribution of chemosensitive and chemoresistant patients in both the training and validation sets is selected to reflect clinical chemosensitive rates of approximately 70% [6]. Similar to the gene expression analysis as stated above, the identification of the morphologic features associated with chemotherapy response was performed using the method described previously [7], [34] in the training set and validated in the validation set: In brief, the signal-to-noise ratio (SNR) is calculated for each potential feature [7], [34], in which positive or negative SNR value indicates the feature favorable for either the chemoresistant or the chemosensitive group. The 153 features are ranked on the basis of their SNR values. The differentially varied morphologic features are determined in the training set on the basis of *FDR*≤2% and then validated in the test set. Feature data exhibit a normal distribution after median centered and log transformed.

### Statistical Analysis

OS and PFS curves are generated by the Kaplan-Meier method, and the statistical significance of survival differences is determined with the log-rank test. Survival analysis is performed and an ROC curve is generated using SPSS (version 17.0; SPSS Inc.) and GraphPad Prism (version 5.04; GraphPad Software, Inc.). Normality of feature values is verified via a Jarque-Bera test [40]. The statistical significance of the morphologic signature is calculated via an unpaired, two-tailed t-test combined with Benjamini-Hochberg (BH) multiple testing [41]. The p-value of the identified pathways is assessed by the Fisher exact test using Ingenuity Pathway Analysis.

## Supporting Information

Figure S1 Overall predictive accuracy of patients in the training set as a function of the gene expression fold change cutoff. The arrow indicates the fold-change cutoff used in the study that gives rise to the highest predictive accuracy.
(PDF)

Figure S2 (A) The average roundness of nuclei in Bin 8 (Mean_Ro_Bin8) is significantly higher in the chemoresistant group (*P* = 1.5 E-04). (B) The same nuclear parameter in Bin 9 (Mean_Ro_Bin9) shows a significant decrease in the chemoresistant group (*P* = 0.0015). (C) The average roundness of the nucleus in an entire sample shows no significant difference between groups (Mean_Ro_Total) (*P* = 0.56).
(PDF)

Figure S3 Overall survival (OS) and progression-free survival (PFS) curves of the 253 patients used for tissue nuclear image profile generation, among which 172 patients were sensitive to chemotherapy and 81 of them were chemoresistant.
(PDF)

Figure S4 Flow chart for nucleus parametric profile generation.
(PDF)

Figure S5 Detailed version of Figure 3A including morphologic feature names.
(PDF)

Table S1 The 227 genes are differentially expressed between chemoresistant and chemosensitive patients (*P*<0.05, as identified by parametric t-test).
(XLS)

Table S2 Clinicopathologic characteristics of patients in the TCGA and Australia Validation data sets.
(PDF)

Table S3 Cox proportional hazard analysis of progression-free survival of OvCa patients in the TCGA and Australian validation data sets in relation to predictive sub-groups (the group with high predictive scores versus the group with low predictive scores).
(PDF)

Table S4 The 15 morphologic features are differentially varied between chemoresistant and chemosensitive patients with serous OvCa (*FDR*≤2%).
(PDF)

Table S5 Image features defined for tumor nuclear image profile generation.
(PDF)

Table S6 Cox proportional hazard analysis of overall and progression-free survival of OvCa patients in relation to the morphological feature value (Std_Ar_Bin2).
(PDF)

Table S7 Significantly expressed gene signatures associated with each morphologic feature.
(PDF)
