# Supplementary material for: Integrated Analysis of Gene Expression and Tumor Nuclear Image Profiles Associated with Chemotherapy Response in Serous Ovarian Carcinoma
Source: PLoS One. 2012 May 8;7(5):e36383. doi: 10.1371/journal.pone.0036383 (PMC3348145; doi:10.1371/journal.pone.0036383)
Supplement: Figure S1 — Overall predictive accuracy of patients in the training set as a function of the gene expression fold change cutoff. The arrow indicates the fold-change cutoff used in the study that gives rise to the highest predictive accuracy. (PDF) [file pone.0036383.s001.pdf]

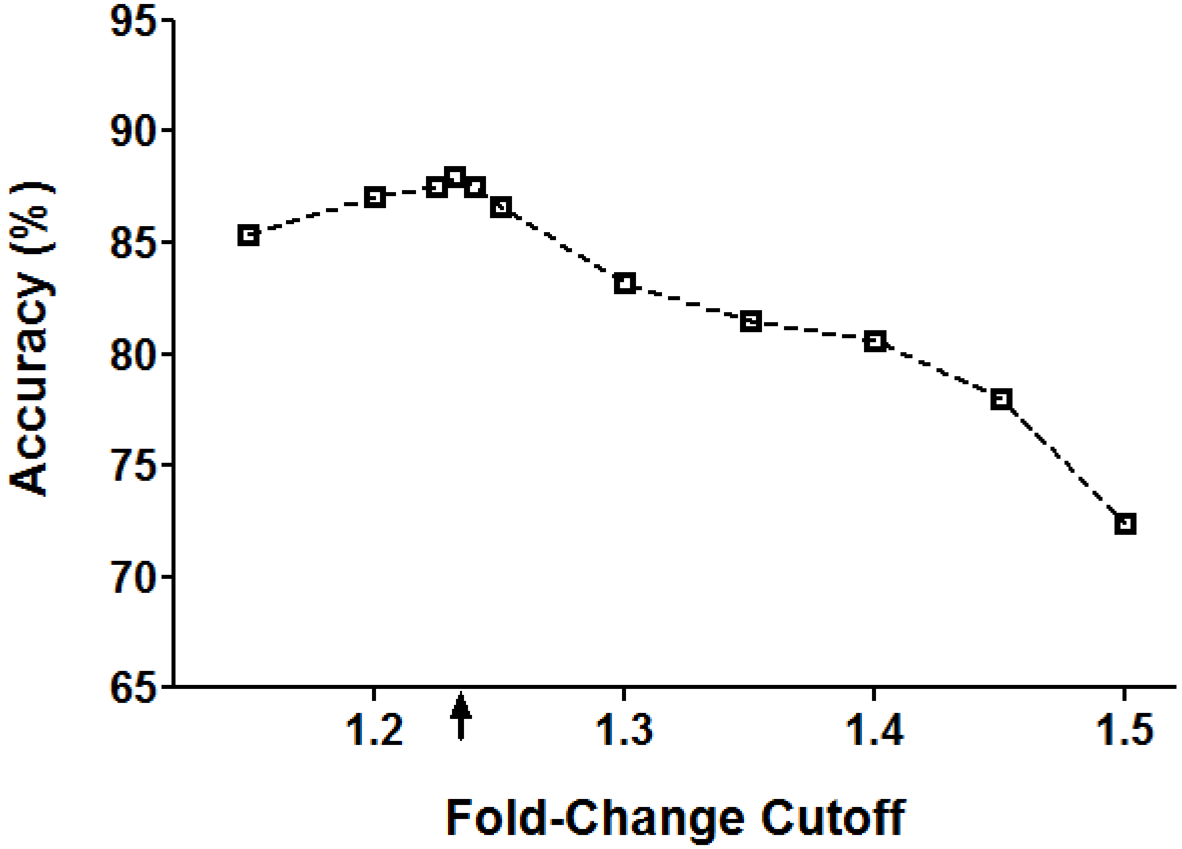


**Figure S1.** Overall predictive accuracy of patients in the training set as a function of the gene expression fold change cutoff. The arrow indicates the fold-change cutoff used in the study that gives rise to the highest predictive accuracy.
