# Supplementary material for: Integrated Analysis of Gene Expression and Tumor Nuclear Image Profiles Associated with Chemotherapy Response in Serous Ovarian Carcinoma
Source: PLoS One. 2012 May 8;7(5):e36383. doi: 10.1371/journal.pone.0036383 (PMC3348145; doi:10.1371/journal.pone.0036383)
Supplement: Figure S2 — (A) The average roundness of nuclei in Bin 8 (Mean_Ro_Bin8) is significantly higher in the chemoresistant group (P = 1.5 E-04). (B) The same nuclear parameter in Bin 9 (Mean_Ro_Bin9) shows a significant decrease in the chemoresistant group (P = 0.0015). (C) The average roundness of the nucleus in an entire sample shows no significant difference between groups (Mean_Ro_Total) (P = 0.56). (PDF) [file pone.0036383.s002.pdf]

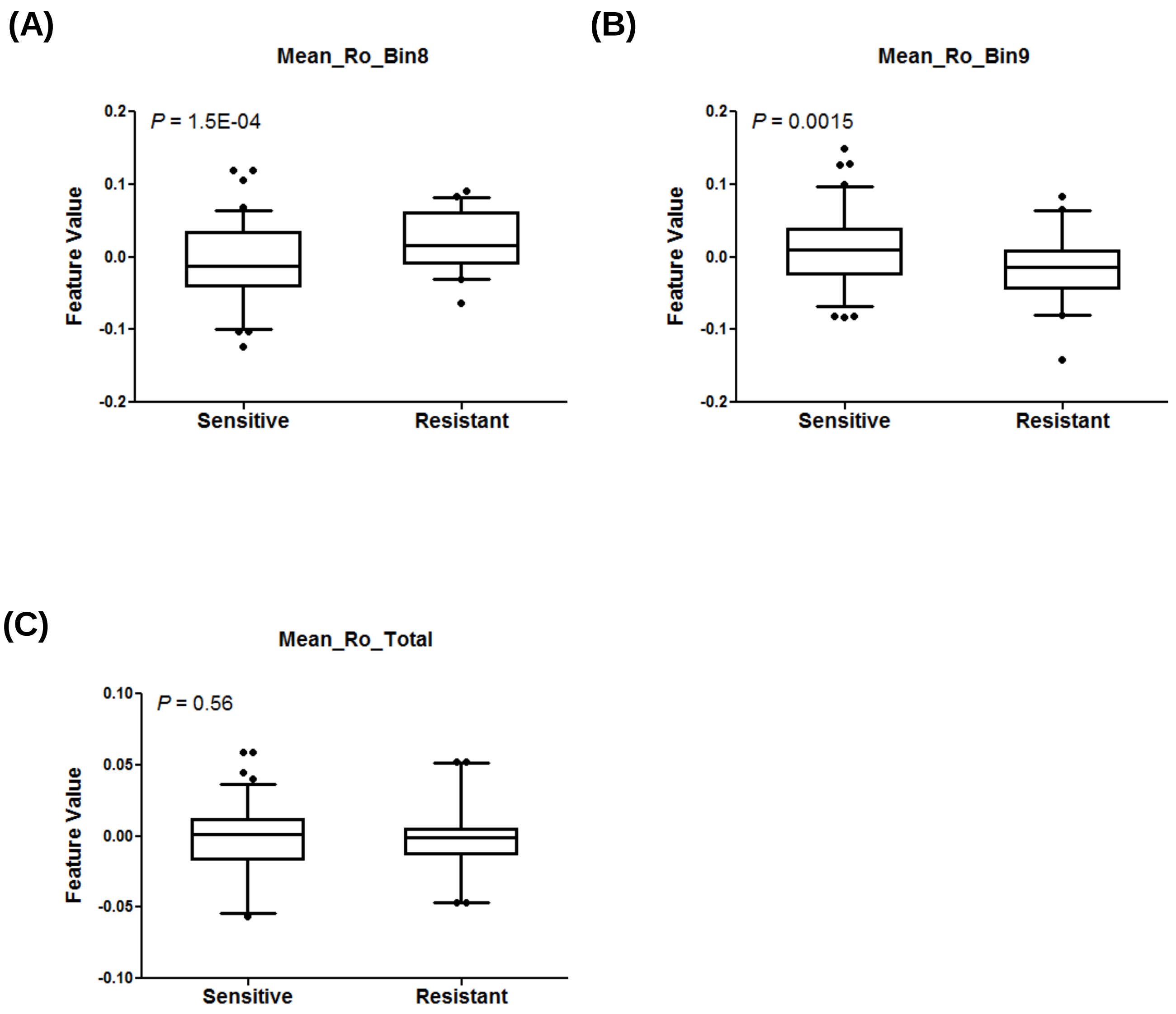


**Figure S2.** (A) The average roundness of nuclei in Bin 8 (Mean_Ro_Bin8) is significantly higher in the chemoresistant group (*P*= 1.5E-04). (B) The same nuclear parameter in Bin 9 (Mean_Ro_Bin9) shows a significant decrease in the chemoresistant group (*P* = 0.0015). (C) The average roundness of the nuclei in an entire sample shows no significant difference between groups (Mean_Ro_Total) (*P* = 0.56).
