# Supplementary material for: Integrated Analysis of Gene Expression and Tumor Nuclear Image Profiles Associated with Chemotherapy Response in Serous Ovarian Carcinoma
Source: PLoS One. 2012 May 8;7(5):e36383. doi: 10.1371/journal.pone.0036383 (PMC3348145; doi:10.1371/journal.pone.0036383)
Supplement: Figure S3 — Overall survival (OS) and progression-free survival (PFS) curves of the 253 patients used for tissue nuclear image profile generation, among which 172 patients were sensitive to chemotherapy and 81 of them were chemoresistant. (PDF) [file pone.0036383.s003.pdf]

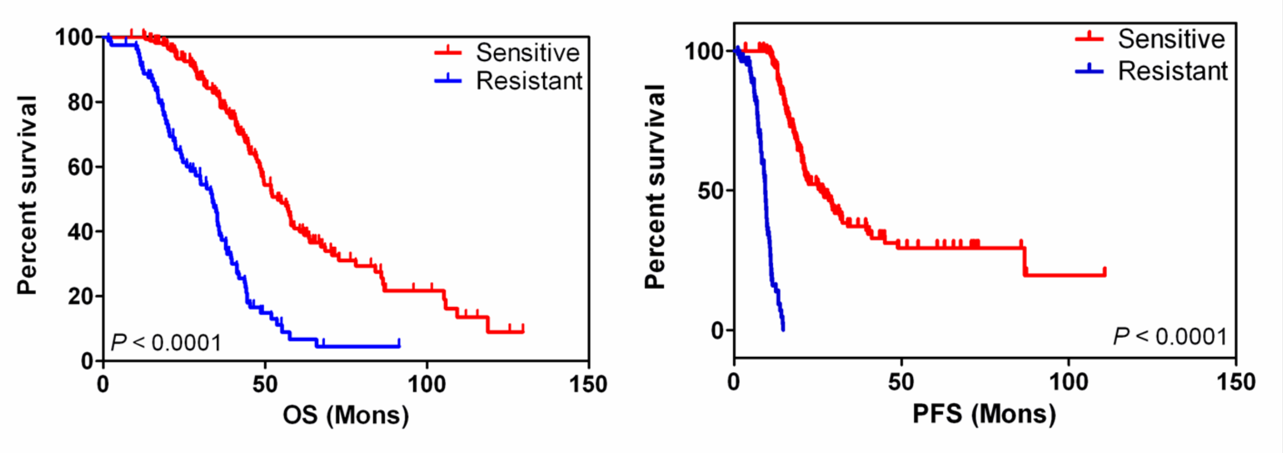

Figure S3. Overall survival (OS) and progression-free survival (PFS) curves of the 253 patients used for tissue nuclear image profile generation, among which 172 patients were sensitive to chemotherapy and 81 of them were chemoresistant.
