# Supplementary figures and images for: Integrated Analysis of Gene Expression and Tumor Nuclear Image Profiles Associated with Chemotherapy Response in Serous Ovarian Carcinoma
Source: PLoS One. 2012 May 8;7(5):e36383. doi: 10.1371/journal.pone.0036383 (PMC3348145; doi:10.1371/journal.pone.0036383)

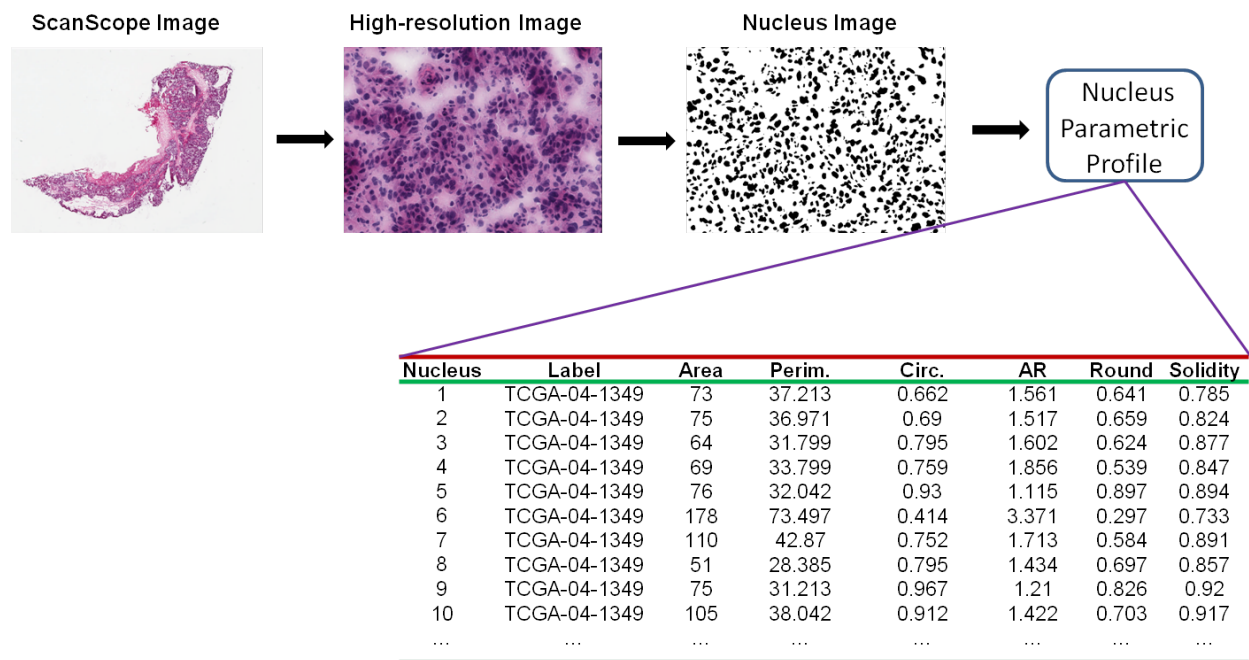

**Figure S4.** Flow chart for nucleus parametric profile generation.

Supplement: Figure S4 — Flow chart for nucleus parametric profile generation. (PDF) [file pone.0036383.s004.pdf]

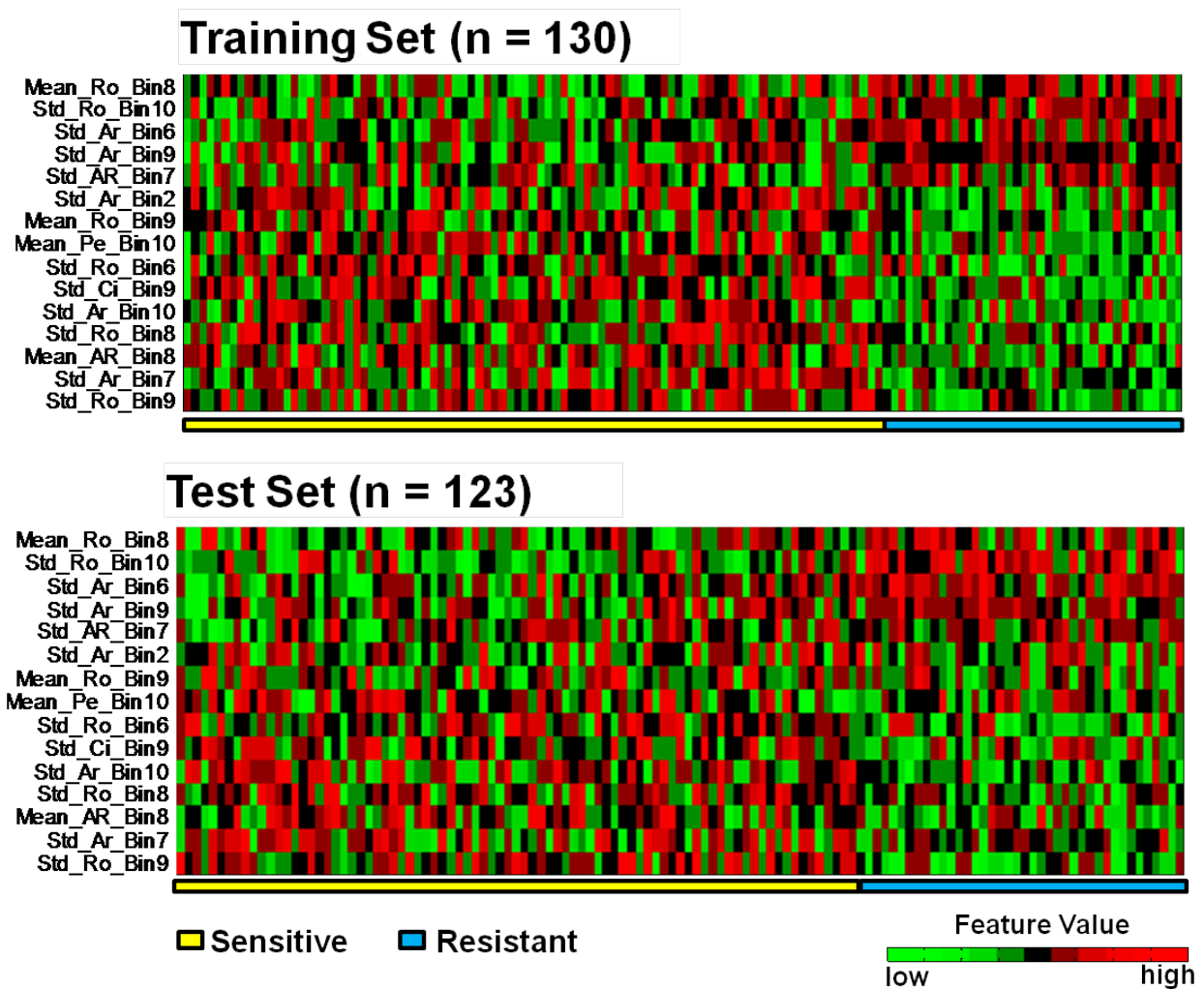

**Figure S5.** Detailed version of Figure 3A including morphologic feature names.

Supplement: Figure S5 — Detailed version of Figure 3A including morphologic feature names. (PDF) [file pone.0036383.s005.pdf]
