# Supplementary material for: Integrated Analysis of Gene Expression and Tumor Nuclear Image Profiles Associated with Chemotherapy Response in Serous Ovarian Carcinoma
Source: PLoS One. 2012 May 8;7(5):e36383. doi: 10.1371/journal.pone.0036383 (PMC3348145; doi:10.1371/journal.pone.0036383)
Supplement: Table S2 — Clinicopathologic characteristics of patients in the TCGA and Australia Validation data sets. (PDF) [file pone.0036383.s007.pdf]

Table S2. Clinicopathologic characteristics of patients in the TCGA and Australia Validation data sets.

	TCGA Valuation Set			Australian Validation Set		
	Group with HS	Group with LS	Totals (All)	Group with HS	Group with LS	Totals (All)
**No. of patients**	185	43	228	171	73	244
**Age**						
**Mean, yrs [SD]**	60.7 [11.2]	59.4 [12.7]	60.5 [11.5]	60.6 [10.4]	59.3 [10.6]	60.2 [10.4]
**Range**	27.2 – 85.5	37.3 – 82.8	27.2 – 85.5	22 - 80	23 - 80	22 - 80
**FIGO Stage**[¶]						
**I**	0	0	0	11	5	16
**II**	9	1	10	9	0	9
**III**	141	33	174	140	58	198
**IV**	31	9	40	11	10	21
**Unknown**	4	0	4	0	0	0
**WHO Grade**						
**2**	15	5	20	59	29	88
**3**	165	37	202	111	43	154
**Unknown**	5	1	6	1	1	2
**Surgical outcome**[ξ]						
**Optimal (≤ 1 cm)**	77	26	103	52	20	72
**Suboptimal (> 1cm)**	46	10	56	42	22	62
**No macroscopic disease**	31	6	37	77	31	108
**Unknown**	31	1	32	0	0	0
**Vital status**						
**Alive**	97	21	118	107	28	135
**Dead**	84	22	106	64	45	109
**Unknown**	4	0	4	0	0	0
**Recurrent disease**[ζ]						
**Yes**	95	27	122	57	11	68
**No**	88	16	104	114	62	176
**Unknown**	2	0	2	0	0	0

Abbreviations: TCGA, The Cancer Genome Atlas; SD, standard deviation; SE, standard error. HS, high-score; LS, low-score

[¶]: Cases were staged according to the 1988 FIGO staging system.

[ξ]: Surgical outcome was defined as the size of residual disease at the conclusion of the primary surgical procedure. This field was used to define surgical cytoreduction as optimal or suboptimal. Optimal was defined as no residual disease greater than 1cm and included the variable categories of no macroscopic disease (*i.e.* microscopic residual disease) and 1 to 10mm. Suboptimal was defined as residual disease greater than 1cm and included the variable categories of 11 to 20mm and greater than 20mm.

[ζ]: Local recurrence after the date of initial surgical resection.
