# Supplementary material for: Integrated Analysis of Gene Expression and Tumor Nuclear Image Profiles Associated with Chemotherapy Response in Serous Ovarian Carcinoma
Source: PLoS One. 2012 May 8;7(5):e36383. doi: 10.1371/journal.pone.0036383 (PMC3348145; doi:10.1371/journal.pone.0036383)
Supplement: Table S3 — Cox proportional hazard analysis of progression-free survival of OvCa patients in the TCGA and Australian validation data sets in relation to predictive sub-groups (the group with high predictive scores versus the group with low predictive scores). (PDF) [file pone.0036383.s008.pdf]

Table S3. Cox proportional hazard analysis of progression-free survival of OvCa patients in the TCGA and Australian validation data sets in relation to predictive sub-groups (the group with higher predictive scores versus the group with lower predictive scores).

	Univariate analysis		Multivariate analysis*	
	Hazard ratio (95% CI)	P_1	Hazard ratio (95% CI)	P_2
TCGA Validation Set	0.501 (0.254, 0.988)	0.046	0.420 (0.196, 0.904)	0.027
Australian Validation Set	0.433 (0.295, 0.635)	1.9×10^{-5}	0.420 (0.283, 0.622)	1.5×10^{-5}

* Adjusted by age, grade and clinical stage.
