# Supplementary material for: Integrated Analysis of Gene Expression and Tumor Nuclear Image Profiles Associated with Chemotherapy Response in Serous Ovarian Carcinoma
Source: PLoS One. 2012 May 8;7(5):e36383. doi: 10.1371/journal.pone.0036383 (PMC3348145; doi:10.1371/journal.pone.0036383)
Supplement: Table S4 — The 15 morphologic features are differentially varied between chemoresistant and chemosensitive patients with serous OvCa (FDR≤2%). (PDF) [file pone.0036383.s009.pdf]

Table S4. The 15 morphologic features are differentially varied between chemoresistant and chemosensitive patients with serous OvCa (*FDR* ≤ 2%).

Features	Description	Feature in Resistant Group (*FDR**)
Std_Ro_Bin9	Standard deviation of roundness in Bin 9	Decreased (1.5E-04)
Std_Ar_Bin7	Standard deviation of area in Bin 7	Decreased (5.4E-03)
Mean_AR_Bin8	Average aspect ratio in Bin 8	Decreased (5.4E-03)
Std_Ro_Bin8	Standard deviation of roundness in Bin 8	Decreased (1.3E-02)
Std_Ar_Bin10	Standard deviation of area in Bin 10	Decreased (1.3E-02)
Std_Ci_Bin9	Standard deviation of circularity in Bin 9	Decreased (1.3E-02)
Std_Ro_Bin6	Standard deviation of roundness in Bin 6	Decreased (1.3E-02)
Mean_Pe_Bin10	Average perimeter in Bin 10	Decreased (1.3E-02)
Mean_Ro_Bin9	Average roundness in Bin 9	Decreased (1.7E-02)
Std_Ar_Bin2	Standard deviation of area in Bin 2	Decreased (1.8E-02)
Std_AR_Bin7	Standard deviation of aspect ration in Bin 7	Increased (2.0E-02)
Std_Ar_Bin9	Standard deviation of area in Bin 9	Increased (1.6E-02)
Std_Ar_Bin6	Standard deviation of area in Bin 6	Increased (5.4E-03)
Std_Ro_Bin10	Standard deviation of roundness in Bin 10	Increased (5.4E-03)
Mean_Ro_Bin8	Average roundness in Bin 8	Increased (5.4E-03)

**FDR* is the feature value difference between the chemosensitive and chemoresistant samples in the training set, as identified by parametric t-test combined with Benjamini-Hochberg multiple testing.
