# Supplementary material for: Integrated Analysis of Gene Expression and Tumor Nuclear Image Profiles Associated with Chemotherapy Response in Serous Ovarian Carcinoma
Source: PLoS One. 2012 May 8;7(5):e36383. doi: 10.1371/journal.pone.0036383 (PMC3348145; doi:10.1371/journal.pone.0036383)
Supplement: Table S5 — Image features defined for tumor nuclear image profile generation. (PDF) [file pone.0036383.s010.pdf]

Table S5. Image features defined for tumor nuclear image profile generation[£].

Features		Area*	Perimeter†	Circularity‡	AR§	Roundness‖	Solidity¶
overall	Mean	√	√	√	√	√	√
	SD	√	√	√	√	√	√
Bin** 1	Mean	√	√	√	√	√	√
	SD	√	√	√	√	√	√
	Percentage††	√	NA	NA	NA	NA	NA
	Count‡‡	√	NA	NA	NA	NA	NA
. . .	. . .	. . .	. . .	. . .	. . .	. . .	. . .
Bin 10	Mean	√	√	√	√	√	√
	SD	√	√	√	√	√	√
	Percentage	√	NA	NA	NA	NA	NA
	Count	√	NA	NA	NA	NA	NA
Compactness§§		√	NA	NA	NA	NA	NA

Abbreviations: AR, aspect ratio; √, feature for the parameter is calculated; SD, standard deviation; NA, feature for the parameter is not applicable.

£: The nuclear parameters such as area, perimeter, circularity, AR, roundness and solidity are automatically measured using ImageJ PlugIns.

*: Area (A) was calculated as the nucleus size in pixels.

†: Perimeter (C) was calculated as the length of the outside boundary of the nucleus.

‡: Circularity was calculated as $4\pi A/C^2$.

§: AR was calculated as major_axis/minor_axis.

‖: Roundness was calculated as $4A/(\pi \times \text{major\_axis}^2)$.

¶: Solidity was calculated as a ratio of the nucleus size to its convex area.

**: Bins were determined based on the nucleus size.

††: Percentage was calculated as a ratio of the number of nuclei in a bin versus in all bins.

‡‡: Count was calculated as the total number of nuclei in a bin.

§§ : Compactness was calculated as a ratio of total number of nuclei to the number of views per sample.
