# Supplementary material for: Integrated Analysis of Gene Expression and Tumor Nuclear Image Profiles Associated with Chemotherapy Response in Serous Ovarian Carcinoma
Source: PLoS One. 2012 May 8;7(5):e36383. doi: 10.1371/journal.pone.0036383 (PMC3348145; doi:10.1371/journal.pone.0036383)
Supplement: Table S6 — Cox proportional hazard analysis of overall and progression-free survival of OvCa patients in relation to the morphological feature value (Std_Ar_Bin2). (PDF) [file pone.0036383.s011.pdf]

Table S6. Cox proportional hazard analysis of overall and progression-free survival of OvCa patients in relation to the morphologic feature value (Std_Ar_Bin2).

	Univariate analysis		Multivariate analysis*	
	Hazard ratio (95% CI)	P_1	Hazard ratio (95% CI)	P_2
Overall survival	0.502 (0.331, 0.763)	0.001	0.453 (0.291, 0.704)	4.3×10^{-4}
Progression-free survival	0.347 (0.141, 0.857)	0.022	0.263 (0.098, 0.705)	0.008

* Adjusted by age, grade and clinical stage.
