# Supplementary material for: Integrated Analysis of Gene Expression and Tumor Nuclear Image Profiles Associated with Chemotherapy Response in Serous Ovarian Carcinoma
Source: PLoS One. 2012 May 8;7(5):e36383. doi: 10.1371/journal.pone.0036383 (PMC3348145; doi:10.1371/journal.pone.0036383)
Supplement: Table S7 — Significantly expressed gene signatures associated with each morphologic feature. (PDF) [file pone.0036383.s012.pdf]

Table S7. Significantly expressed gene signatures associated with each of morphologic features.

Morphologic Signatures	Differentially Expressed Gene Signatures											
Std_Ro_Bin9	MATK	IL15	CREB5	HSD11B2	MDS1	NAP5	LINGO1	EVI1	VTCN1	KCNN3	GPT2	
Std_Ar_Bin7	LRIG1	ABP1	SUSD4	MAGEE2								
Mean_AR_Bin8	CPEB1	SETBP1	NFIB	PHGDH	LINGO1	C1orf115	SYT13					
Std_Ro_Bin8	FOXA2	PCSK6	RAB27B									
Std_Ar_Bin10	FMN2	HSD11B2	IL11RA	C22orf36	EPHB3	TMTC1	RTN4R	LINGO1	NFIB	WIT1		
Std_Ci_Bin9	GAP43											
Std_Ro_Bin6	BCHE	DIRC1	IL27	MAB21L2	CRCT1	PRSS36	C6orf124	ODZ3	SLC26A9			
Mean_Pe_Bin10	SPANXD	BEST4	ISG20	MDS1								
Mean_Ro_Bin9	IL15	SV2C	PSAT1	BTNL9								
Std_Ar_Bin2	CREB5	SCGB3A1	KCNB1	NPR3	IGFBP5	KCNN3						
Std_AR_Bin7	C1orf115											
Std_Ar_Bin9	XBP1	SAPS1	C9orf47									
Std_Ar_Bin6	HMGCS1	LSM8	CXCR4	LIPG	FAM19A4	MMP17						
Std_Ro_Bin10	SLC5A1	B3GALNT1	CHIT1	XBP1	UGT8	TTYH1	AREG	FLJ21963	TRIM49	EXTL1	ODZ3	NPY
Mean_Ro_Bin8	FZD4	SLC1A3	PHGDH	NKX6-2								
